# Dyslipidemia Is Related to Mortality in Critical Patients With Coronavirus Disease 2019: A Retrospective Study

**DOI:** 10.3389/fendo.2021.611526

**Published:** 2021-06-23

**Authors:** Jiang Yue, Hua Xu, Yong Zhou, Wen Liu, Xiaofeng Han, Qing Mao, Shengxian Li, Lai-Shan Tam, Jing Ma, Wei Liu

**Affiliations:** ^1^ Department of Endocrinology and Metabolism, Renji Hospital, School of Medicine, Shanghai Jiao Tong University, Shanghai, China; ^2^ Department of Cardiology, Renji Hospital, School of Medicine, Shanghai Jiao Tong University, Shanghai, China; ^3^ Department of Hematology, Renji Hospital, School of Medicine, Shanghai Jiao Tong University, Shanghai, China; ^4^ Department of Neurosurgery, Renji Hospital, School of Medicine, Shanghai Jiao Tong University, Shanghai, China; ^5^ Department of Medicine and Therapeutics, The Prince of Wales Hospital, The Chinese University of Hong Kong, Hong Kong, Hong Kong

**Keywords:** coronavirus disease 2019 (COVID-19), critical patients, lipid metabolism, mortality, risk factor

## Abstract

**Background:**

It has been reported that dyslipidemia is related to coronavirus-related diseases. Critical patients with coronavirus disease 2019 (COVID-19) who suffered from multiple organ dysfunctions were treated in the intensive care unit (ICU) in Wuhan, China. Whether the lipids profile was associated with the prognosis of COVID-19 in critical patients remained unclear.

**Methods:**

A retrospective study was performed in critical patients (N=48) with coronavirus disease 2019 in Leishenshan hospital between February and April 2020 in Wuhan. The parameters including lipid profiles, liver function, and renal function were collected on admission day, 2-3days after the admission, and the day before the achievement of clinical outcome.

**Results:**

Albumin value and creatine kinase (ck) value were statistically decreased at 2-3 days after admission compared with those on admission day (*P*<0.05). Low density lipoprotein (LDL-c), high density lipoprotein (HDL-c), apolipoprotein A (ApoA), and apolipoprotein A (Apo B) levels were statistically decreased after admission (*P*<0.05). Logistic regression showed that HDL-c level both on admission day and the day before the achievement of clinical outcome were negatively associated with mortality in critical patients with COVID-19. Total cholesterol (TC) level at 2-3days after admission was related to mortality in critical patients with COVID-19.

**Conclusions:**

There were lipid metabolic disorders in the critical patients with COVID-19. Lower levels of HDL-c and TC were related to the progression of critical COVID-19.

## Introduction

At the end of 2019, the outbreak of pneumonia was later named as 2019 novel coronavirus (2019-nCoV) or coronavirus disease 2019 (COVID-19) by the World Health Organization (WHO) (1). COVID-19 is caused by SARS-Cov-2, which mainly invades the respiratory tract leading to acute respiratory distress syndrome (ARDS), septic shock, and multiple organ dysfunction syndromes (MODS) (2). The prevalence of death is about 2.3% between 6 to 41 days after symptom onset (3, 4). The risk factors of mortality in patients with severe COVID-19 include older age and pre-existing conditions, such as cardiovascular diseases, cancer, acute kidney disease, and diabetes mellitus (5, 6).

It has been reported that dyslipidemia was related to coronavirus-related diseases. Hypolipidemia could be acquired by multiple diseases, which include cancers (colorectal, prostatic carcinoma, leukemias, myeloma, and other monoclonal gammopathies), malabsorption, anemia, and severe illness (7). Abnormal lipid metabolism was observed in the recovered SARS patients 12 years after infection (8). Recently, a retrospective study indicated that decreased low density lipoprotein (LDL) levels may be a potential predictor of poor prognosis in patients with COVID-19 (9). Therefore, we aimed to investigate the lipid profiles in critical patients with COVID-19 and its link with mortality.

## Materials and Methods

### Study Design and Patients

This was a retrospective study carried out in Leishenshan Hospital, Wuhan, China. A total number of 48 adult critical patients (≥18 years old) with COVID-19 who were hospitalized at two intensive care units (ICUs) of Leishenshan Hospital, were observed in this study. All the patients were admitted to ICUs from February 22 to March 31, 2020, and were either discharged or died by April 6, 2020. The study protocol was conducted according to the Declaration of Helsinki. The study has been approved by the Medical Ethics Committee of Renji Hospital, School of Medicine, Shanghai Jiaotong University. Electronic medical data including demographic, diagnosis, clinical treatment, laboratory parameters were exacted. Pneumonia was diagnosed based on the guidelines from the Chinese Thoracic Society and the Chinese Medicine Association. In total, 48 critical patients with COVID-19 were enrolled. They were wholly transferred from general wards or other hospitals in Wuhan if they all fulfilled the following criteria: patients with laboratory-confirmed COVID-19, who diagnosed by quantitative reverse transcription-polymerase chain reaction (RT-qPCR), were eligible if they met the following criteria: (1) respiratory rate ≥30 breaths/min; (2) pulse oxygen saturation (SP02) rate ≤ 93% at rest; (3) patients who had severe pneumonia with more than 50% of progression in the extent of chest CT abnormalities within 48 h; (4) PAO_2_/FIO_2_of < 300 mmHg; and (5) patients who had been supported by mechanical ventilation. Patients were excluded if (1) the result of the COVID-19 nucleic acid amplification test was negative; (2) patients met the diagnostic criteria of COVID-19, while they suffered from other diseases, such as acute myocardial infarction, acute decompensated heart failure, and chronic liver disease; (3) patients without respiratory failure (10, 11).

Patients with laboratory-confirmed COVID-19 infection who had any of the following items were considered to be in critical conditions: (1) respiratory failure indicating the necessity of mechanical ventilation; (2) septic shock patients who were identified by the use of vasopressor therapy and elevated lactate levels (>2 mmol/L) despite adequate fluid resuscitation, or (3) failure of other organs requiring admission to the ICU (10, 11).

### Clinical Laboratory Parameters

All the tests were performed in the certified clinical laboratory of Leishenshan Hospital under standard procedures and practices that fully complied with the regulations and guidelines of the Chinese Food and Drug Admission and Center for Disease Control. Blood samples were collected at 8:00 a.m. after 8-12 hours of fasting. The blood samples were immediately sent to the laboratory of Leishenshan Hospital in Wuhan. After centrifugation, the samples were tested for blood routine, blood glucose, blood lipid profiles, liver and renal function, blood calcium, blood phosphorus, and blood magnesium as well as other parameters. Biochemical indexes such as blood glucose, blood lipid profiles as well as liver and renal function, were measured by a Roche Cobas 8000 automatic biochemical analyzer. The clinical laboratory data in this study were collected at three time points, including on the first day of admission day, 2-3 days after the admission, and on the day before achievement of clinical outcome (died or discharged).

### Statistical Analysis

Data were expressed as mean ± standard deviation (SD) or median (interquartile range [IQR]) for numerical variables. Differences in the laboratory parameters between groups were assessed by either the Mann-Whitney U test or χ2 test. A Chi-squared test was used to compare categorical variables between groups. The parameters in terms of glucose, liver function, renal function, inflammation factors, serum calcium, phosphorus, magnesium as well as lipid profiles on admission, day 2-3 after admission, and last test before achievement of clinical outcome were evaluated by repeated measures one-way ANOVA. Logistic regression analysis was performed to identify the independent variables associated with in-hospital mortality, including demographic- and disease-specific variables as well as lipid profile and so on. Statistical analyses were performed by SPSS 22.0 (SPSS, Chicago, IL, USA) and a two-sided *P* < 0.05 was considered statistically significant.

## Results

### Demographic and Clinical Characteristics of COVID-19 Patients

A total number of 48 critical patients with COVID-19 were recruited in this study. The median (IQR) age of all the critical patients was 68 (62,78) years. There were more male patients than female patients (67% &33%). 18 critical patients did not survive with a mortality rate of 37.5% (18/48). The proportion of patients with comorbidities including type 2 diabetes mellitus (T2DM), hypertension, cardiovascular disease (CVD), and stroke were 14.6%, 27.1%, 12.5%, and 10.4% respectively (**Table 1**).

**Table 1:** Demographic and clinical characteristics of critical patients with COVID-19.

Characteristic	Total (N=48)	Deceased (N=18)	Survived (N=30)	*P* value
Age (years)	68 (62,78)	73 (63,81)	67 (62,77)	0.233
Male	32 (66.7%)	12	20	1.000
Female	16 (33.3%)	6	10	
Comorbidities				
T2DM	7 (14.6%)	6 (33.3%)	1 (3.33%)	0.008
Hypertension	13 (27.1%)	7 (38.9%)	6 (20%)	0.154
CVD	6 (12.5%)	3 (16.7%)	3 (10%)	0.658
Stroke	5 (10.4%)	3 (16.7%)	2 (6.67%)	0.349
Life-support treatment				
Oxygen	48 (100%)	18 (100%)	30 (100%)	
Mechanical Ventilation	33 (68.8%)	18 (100%)	15 (50%)	0.000

T2DM, Type 2 diabetes mellitus; CVD, cardiovascular diseases.

Data expressed as median ((interquartile range [IQR])) or number (percentage).

All the recruited patients were not given statin therapy due to the low LDL level and critical status. The patients with hypertension on admission were given the same anti-hypertension drug, such as ACEI or ARB if they were conscious. If they were unconscious, they were given calcium channel blockers or nitrates according to the blood pressure level and cardiovascular status. Heparin was applied if the D-dimer level was high. No statistical differences existed between the surviving patients and deceased patients in terms of the application of ACEI or ARB or heparin therapy (*p*>0.05).

Patients with T2DM had a significantly higher mortality rate (*p*<0.05, **Table 1**), indicating that T2DM is a potential poor prognostic factor for COVID-19. Clinical laboratory tests showed that no significant differences existed between the groups who died and those who survived on the day of admission, day 2-3 after admission, and last test before achievement of clinical outcome group in terms of glucose level, liver function, renal function and ion concentration (*p*>0.05, **Table 2**).

**Table 2:** Main clinical laboratory tests of critical patients with COVID-19.

Laboratory profiles	On admission (point 1)	Day 2-3 after admission (point 2)	Last test before achievement before clinical outcome (point 3)	*P* value (point 1&point 2)	*P* value (point 1&point 3)	*P* value (point 2&point 3)
Glucose (mmol/L)	7.36 ± 3.87	7.93 ± 4.14	8.61 ± 4.13	1.000	0.146	1.000
Cre (umol/L)	129.14 ± 183.84	122.68 ± 147.36	138.13 ± 156.81	1.000	1.000	0.622
UA (umol/L)	264.79 ± 146.79	250.79 ± 135.74	239.55 ± 184.62	0.688	1.000	1.000
Cys (mg/L)	1.66 ± 1.19	1.62 ± 1.14	1.79 ± 1.34	1.000	0.860	0.204
PCT (ng/mL)	3.22 ± 11.97	2.46 ± 10.00	1.53 ± 2.72	0.285	1.000	1.000
ALT (IU/L)	54.29 ± 88.57	58.06 ± 83.89	115.28 ± 293.60	1.000	0.795	0.875
AST (IU/L)	64.36 ± 99.65	51.78 ± 53.65	205.59 ± 554.47	0.983	0.513	0.395
TP (g/L)	59.03 ± 7.79	56.62 ± 8.25	54.92 ± 8.21	0.015	0.032	0.682
ALB (g/L)	30.58 ± 3.68	29.18 ± 4.03	30.47 ± 5.68	0.010	1.000	0.538
TBIL (umol/L)	14.04 ± 10.57	14.35 ± 11.63	17.46 ± 17.64	1.000	0.812	0.888
DBIL (umol/L)	7.87 ± 6.65	8.28 ± 8.62	12.81 ± 16.99	1.000	0.261	0.274
ALP (IU/L)	83.40 ± 34.55	87.06 ± 39.44	95.26 ± 41.14	1.000	0.183	0.623
GGT (IU/L)	47.18 ± 35.24	53.55 ± 42.92	52.55 ± 38.31	0.368	1.000	1.000
K^+^ (mmol/L)	4.43 ± 0.76	4.33 ± 0.71	4.52 ± 0.65	1.000	1.000	0.921
Na^+^ (mmol/L)	140.50 ± 4.55	141.02 ± 6.58	142.91 ± 7.25	1.000	0.160	0.596
C-Ca^2+^ (mmol/L)	2.17 ± 0.11	2.19 ± 0.14	2.24 ± 0.12	0.689	0.006	0.203
P (mmol/L)	0.98 ± 0.37	0.91 ± 0.33	1.06 ± 0.63	0.441	0.985	0.384
Mg^2+^ (mmol/L)	0.85 ± 0.10	0.85 ± 0.12	0.86 ± 0.17	1.000	1.000	1.000
CK (IU/L)	537.21 ± 1090.04	348.42 ± 727.26	247.13 ± 443.20	0.042	0.247	0.954
LDH (IU/L)	445.28 ± 182.38	456.52 ± 260.09	591.97 ± 714.84	1.000	0.807	0.946
HBDH (IU/L)	354.13 ± 155.58	352.55 ± 189.34	359.29 ± 243.26	1.000	1.000	1.000
TC (mmol/L)	3.03 ± 1.21	2.88 ± 1.12	2.44 ± 1.21	0.773	0.062	0.076
TG (mmol/L)	0.82 ± 0.52	0.93 ± 0.62	1.09 ± 0.67	0.867	0.213	0.875
HDL (mmol/L)	0.93 ± 0.35	0.86 ± 0.34	0.63 ± 0.28	0.172	0.000	0.004
Non-HDL (mmol/L)	2.03 ± 0.21	1.97 ± 0.19	1.79 ± 0.20	0.637	0.259	0.253
LDL (mmol/L)	2.00 ± 0.66	1.90 ± 0.65	1.54 ± 0.88	0.424	0.012	0.034
APOA (g/L)	0.90 ± 0.25	0.84 ± 0.28	0.64 ± 0.31	0.90	0.001	0.009
APOB (g/L)	0.86 ± 0.25	0.84 ± 0.24	0.65 ± 0.30	1.000	0.009	0.008

Cre, creatinine; UA, uric acid; Cys, Cystatin; PCT, Procalcitonin; ALT, Alanine aminotransferase; AST, Glutamic oxaloacetylase; TP, Total protein; ALB, Albumin; TBIL, total bilirubin; DBIL, Direct bilirubin; ALP, alkaline phosphatase; GGT, r-glutamyltranspeptidase; C-Ca2+ corrected calcium; LDH, lactate dehydrogenase; HBDH, α-Hydroxybutyrate dehydrogenase; TC, total cholesterol; TG, triglyceride; HDL, high density lipoprotein; LDL, low density lipoprotein; APOA, apolipoprotein A; APOB, apolipoprotein B.

Data expressed as mean ± standard deviation (SD).

### Lipid Profiles in Critical Patients With COVID-19

**Figure 1:**
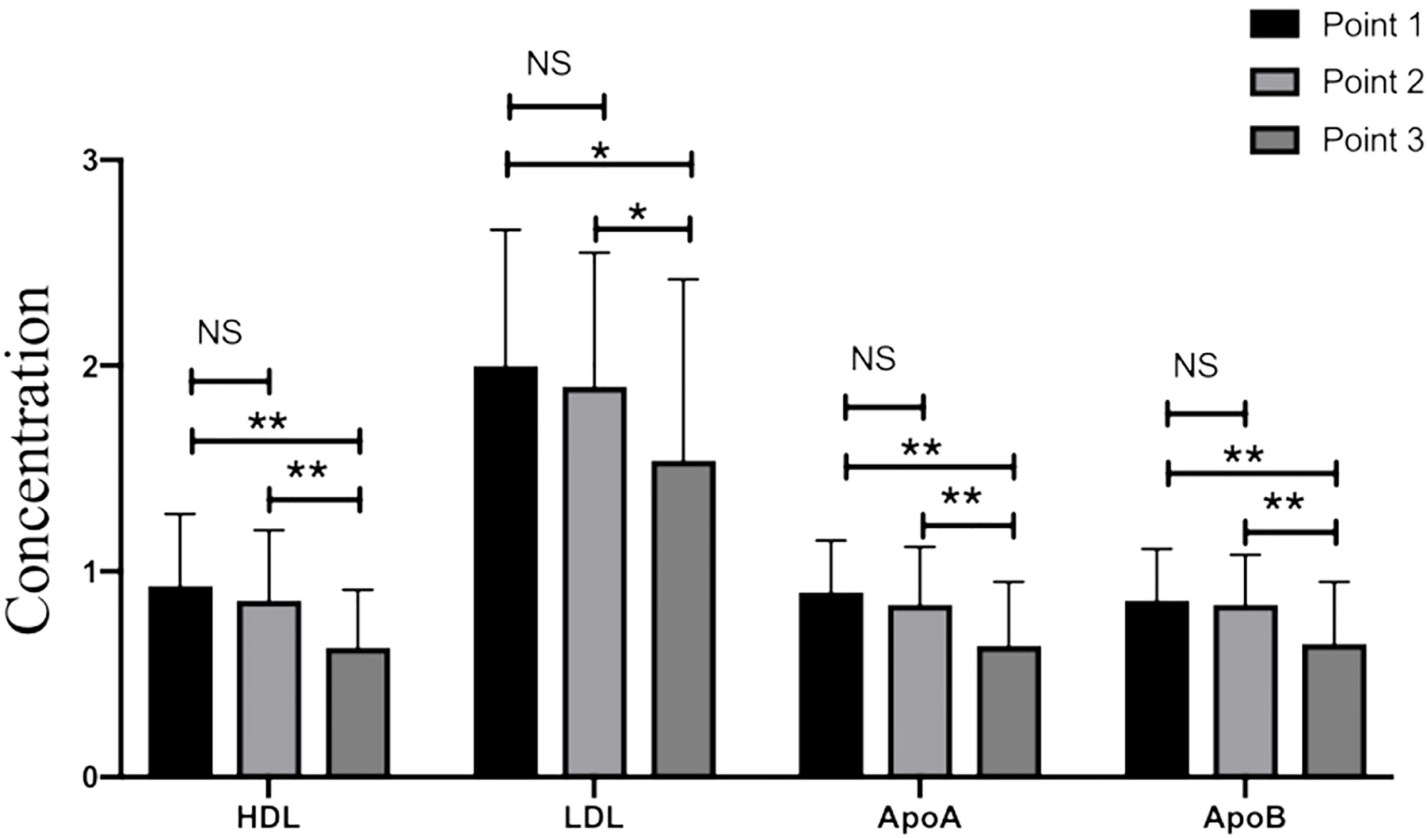
The levels of HDL-c, LDL-c, ApoA, and ApoB in critical patients with COVID-19. Point 1: on admission day; Point 2: day 2-3 after the admission; Point 3: last test before achievement of clinical outcome. NS: *p* > 0.05; *: *p* < 0.05; **: *p* < 0.01.

The levels of HDL-c, LDL-c, Apolipoprotein A (ApoA), and Apolipoprotein B (ApoB) were statistically lower on the day before the achievement of clinical outcome group compared with those in the admission group (*p*<0.05, **Table 2** and **Figure 1**). Meanwhile, significant differences still existed between day 2-3 after admission group and last test before achievement of clinical outcome group in terms of HDL-c, LDL-c, ApoA, and ApoB levels (*p*<0.05, **Table 2** and **Figure 1**). No significant differences were found in triglycerides (TG), TC level as well as non-HDL between the above three time-points (*p*>0.05, **Table 2**).

Then we divided the 48 critical patients with COVID-19 into two groups according to the achievement of clinical outcomes: deceased group (group 1) and survival group (group 2). The lipid profiles were compared between the two groups at the three time-points (TP). The levels of HDL-c, TC, and ApoA were statistically lower in group 1 than those in group 2 at TP-1 (on admission), and the above parameters were still significantly lower in group 1 compared with those in group 2 at TP-3 (before achievement of clinical outcome) (*p*<0.05, **Table 3**). Meanwhile, TC level was much higher in group 2 compared with that in group 1 at TP-2 (day 2-3 after admission) (*p*<0.05, **Table 3**). There was no significant difference in LDL-c level between the two groups (*p*=0.054, **Table 3**). LDL-c levels were lower in group 1 than group 2 at TP-3 (*p*<0.05, **Table 3**).

**Table 3:** Main clinical laboratory profiles of critical patients with COVID-19.

Laboratory tests	On admission				Day2-3 after admission				Last test before achievement of clinical outcome			
	Total (N=48)	Deceased (N=18)	Survivial (N=30)	P value	Total (N=48)	Deceased (N=18)	Survivial (N=30)	P value	Total (N=48)	Deceased (N=18)	Survivial (N=30)	P value
Glucose (mmol/L)	6.5(5.4, 9.1)	7.24(5.41,11.39)	6.49(4.72,8.69)	0.688	6.62 (4.77, 9.19)	7.52(6.05,9.02)	6.33(4.65,9.20)	0.546	7.07 (5.31, 11.53)	7.96(6.02,15.54)	6.89(5.07, 10.63)	0.180
Cre (umol/L)	73 (55, 110)	80.70(60.50,121.25)	68.40(51.10,99.10)	0.289	75.05 (51.30,144.20)	81.50(57.90,127.70)	73.4(50.65,157.75)	0.618	74.4 (49.88,159.83)	118.10(55.30,219.20)	73.20(47.75,125.30)	0.363
UA (umol/L)	230 (164, 332)	225(153.50,335.50)	235.00(161.00,333.00)	0.664	231 (150, 313)	168(127.75,264.25)	242.00(191.50,331.00)	0.130	160 (124, 308)	151(90,354.50)	163.00(124.50,300.50)	0.559
Cys (mg/L)	1.21 (1.03,1.85)	1.28 (1.09,2.10)	1.18 (0.93,1.71)	0.339	1.15 (1.00, 2.14)	1.22 (1.07,1.95)	1.14 (0.95,2.16)	0.511	1.30 (0.97, 2.51)	1.62 (1.07,2.69)	1.05 (0.89,1.91)	0.201
PCT (ng/mL)	0.22 (0.10,1.21)	1.10 (0.19,1.78)	0.13 (0.08,0.30)	0.033	0.21 (0.09, 0.62)	0.39 (0.17,0.79)	0.21 (0.088,0.57)	0.473	0.30 (0.11,1.30)	1.03 (0.61,4.79)	0.14 (0.08,1.08)	0.017
ALT (IU/L)	30 (18, 48)	35 (24.40,61.05)	25.00 (17.00,48.50)	0.162	37.50 (17.75, 65.50)	25.50 (17.75,67.25)	39 (17.25,64.50)	0.677	28.00 (12.00, 56.50)	35 (4.75,228.00)	25.00 (26.00,58.75)	0.681
AST (IU/L)	38 (23, 58)	43 (38,67)	30.00 (21.00,49.50)	0.042	40.00 (26.75, 59.25)	38.50 (31.50,82.00)	40.5 (26.00,58.75)	0.532	33.00 (21.50, 60.00)	58 (29.75,403.75)	24.00 (20.00,35.00)	0.024
TP (g/L)	58 (53, 63)	58.10 (50.85,62.95)	58.30 (53.65,63.50)	0.724	57.15 (50.10, 63.05)	54.10 (46.73,59.23)	59.30 (50.40,64.30)	0.151	54.00 (50.40, 61.20)	51.50 (45.78,59.83)	54.90 (50.50,65.20)	0.096
ALB (g/L)	30 (27, 32)	28.55 (24.08,32.35)	30.20 (28.55,32.40)	0.125	28.7 (27.15, 31.00)	28 (23.55,30.18)	28.90 (27.43,32.38)	0.112	29.90 (27.60, 33.40)	29.50 (23.40,31.58)	30.80 (28.10,35.00)	0.092
TBIL (umol/L)	11.6 (7.4, 17.8)	14.20 (7.23,23.85)	10.75 (7.25,15.95)	0.237	11.40 (7.15, 17.50)	11.75 (7.68,27.38)	11.40 (6.65,17.00)	0.545	10.10 (7.10, 20.15)	24.50 (13.33,46.45)	8.20 (6.90,14.90)	0.000
DBIL (umol/L)	5.8 (4.0, 9.9)	8.20 (4.78,15.35)	5.10 (3.38,7.40)	0.053	4.95 (3.68, 10.30)	7.15 (4.35,16.95)	4.65 (3.10,9.95)	0.219	89.50 (61.00, 120.00)	19.80 (8.30,39.25)	4.50 (2.80,10.00)	0.001
ALP (IU/L)	85.5 (60.5, 118)	97 (69.75,142.15)	82.50 (57.50,112.75)	0.265	77.00 (60.50, 103.0)	64 (59,84.50)	83.00 (62.00, 112.0)	0.273	89.50 (61.00,120.00)	77.00 (56.25,116.25)	92.00 (66.75,123.50)	0.528
GGT (IU/L)	46 (29,76)	55.95 (25,117.75)	39.00 (29.00,62.50)	0.283	46.00 (29.00, 69.00)	36.50 (14.00,80.50)	47.00 (30.00,66.00)	0.531	42.00 (30.50, 58.25)	41.00 (18.75,62.75)	43.50 (32.75,60.50)	0.655
K^+^ (mmol/L)	4.4 (3.8, 4.8)	4.48 (3.85,4.82)	4.40 (3.75,4.77)	0.753	4.26 (3.88, 4.67)	4.11 (3.85,4.38)	4.39 (3.80,5.16)	0.243	4.44 (4.05,4.97)	5.07 (4.44,5.50)	4.27 (3.86,4.68)	0.017
Na^+^ (mmol/L)	140 (137.3, 145.7)	145.75 (139.63,148.10)	139.30 (136.90,140.90)	0.003	140.4 (137.6,144.08)	143.65 (134.73,151.33)	140.05 (137.60,142.83)	0.267	140.85 (136.93,147.9)	141.85 (137.50,153.10)	140.85 (136.33,145.65)	0.408
C-Ca^2+^ (mmol/L)	2.17 (2.10, 2.25)	2.17 (2.10,2.24)	2.16 (2.10,2.28)	0.633	2.19 (2.13,2.26)	2.22 (2.14,2.26)	2.18 (2.06,2.28)	0.610	2.23 (2.12,2.31)	2.22 (2.12,2.33)	2.24 (2.06,2.28)	1.000
P (mmol/L)	0.92 (0.79, 1.19)	0.98 (0.79,1.22)	0.85 (0.78,1.17)	0.364	0.83 (0.69,1.06)	0.69 (0.53,0.92)	0.86 (0.77,1.14)	0.058	0.93 (0.71,1.25)	0.89 (0.58,1.68)	0.95 (0.75,1.22)	0.715
Mg^2+^ (mmol/L)	0.84 (0.77, 0.95)	0.91 (0.81,1.04)	0.84 (0.77,0.89)	0.060	0.82 (0.78,0.93)	0.86 (0.75,0.96)	0.82 (0.78,0.92)	0.705	0.83 (0.76,0.90)	0.84 (0.77,0.97)	0.82 (0.76,0.87)	0.514
CK (IU/L)	96 (42, 429)	186 (59.05,1266.25)	78.00 (38.00,192.00)	0.191	103 (40.50,326.75)	323.00 (90.50,458.50)	75.00 (33.00,166.00)	0.034	72 (32.5,143.75)	159.00 (111.50,856.50)	52.00 (29.00,87.00)	0.006
LDH (IU/L)	407 (283, 528)	447.90 (366.00,644.25)	376.00 (256.00,477.00)	0.128	401.0 (277.5,545.5)	449.00 (372.5,593.00)	380.00 (224.00,544.00)	0.187	324.50 (257.00,602.75)	645.00 (432.00,1279.00)	276.00 (229.00,369.00)	0.002
HBDH (IU/L)	346 (214, 455)	407.50 (313.50,576.00)	305.00 (192.00,364.00)	0.017	312.5 (216.8,429.75)	372.00 (280.00,439.50)	290.00 (182.00,429.00)	0.285	255.00 (189.25,467.75)	395.00 (322.00,647.00)	216.00 (176.00,278.00)	0.004
TC (mmol/L)	3.07 (2.10, 3.67)	2.56 (2.04,3.25)	3.31 (2.55,4.01)	0.029	2.82 (2.37,3.53)	2.32 (1.47,2.62)	3.13 (2.67,3.88)	0.002	2.18 (1.68,2.89)	1.69 (1.34,2.22)	2.29 (1.76,3.77)	0.037
TG (mmol/L)	0.85 (0.56, 1.32)	0.93 (0.64,1.41)	0.81 (0.43,1.17)	0.257	0.94 (0.58,1.18)	0.71 (0.20,1.21)	0.96 (0.74,1.13)	0.377	0.91 (0.61,1.30)	0.97 (0.80,1.40)	0.83 (0.57,1.27)	0.248
HDL (mmol/L)	0.81 (0.67, 1.03)	0.75 (0.64,0.88)	0.90 (0.69,1.29)	0.017	0.78 (0.68,0.96)	0.76 (0.63,0.87)	0.83 (0.69,1.03)	0.238	0.61 (0.43,0.88)	0.49 (0.27,0.62)	0.69 (0.46,0.93)	0.039
LDL (mmol/L)	1.79 (1.31, 2.18)	1.69 (1.15,2.08)	1.92 (1.46,2.55)	0.136	1.74 (1.48,2.08)	1.46 (1.24,1.98)	1.84 (1.64,2.15)	0.054	1.33 (1.00,1.96)	1.06 (0.71,1.32)	1.57 (1.13,2.26)	0.015
APOA (g/L)	0.81 (0.68, 1.05)	0.75 (0.63,0.86)	0.87 (0.71,1.23)	0.033	0.79 (0.69,1.01)	0.73 (0.64,0.82)	0.85 (0.69,1.08)	0.144	0.60 (0.43,0.89)	0.43 (0.28,0.56)	0.76 (0.56,1.00)	0.002
APOB (g/L)	0.81 (0.62, 1.05)	0.83 (0.64,1.02)	0.81 (0.59,1.10)	0.776	0.75 (0.68,0.97)	0.74 (0.63,0.97)	0.79 (0.68,0.97)	0.479	0.62 (0.50,0.79)	0.50 (0.40,0.67)	0.65 (0.52,0.89)	0.094

Cre, creatinine; UA, uric acid; Cys, Cystatin; PCT, Procalcitonin; ALT, Alanine aminotransferase; AST, Glutamic oxaloacetylase; TP, Total protein; ALB, Albumin; TBIL, total bilirubin; DBIL, Direct bilirubin; ALP, alkaline phosphatase; GGT, r-glutamyltranspeptidase; C-Ca2+ corrected calcium; LDH, lactate dehydrogenase; HBDH, α-Hydroxybutyrate dehydrogenase; TC, total cholesterol; TG, triglyceride; HDL, high density lipoprotein; LDL, low density lipoprotein; APOA, apolipoprotein A; APOB, apolipoprotein B.Data expressed as median (interquartile range [IQR]).

**Figure 2:**
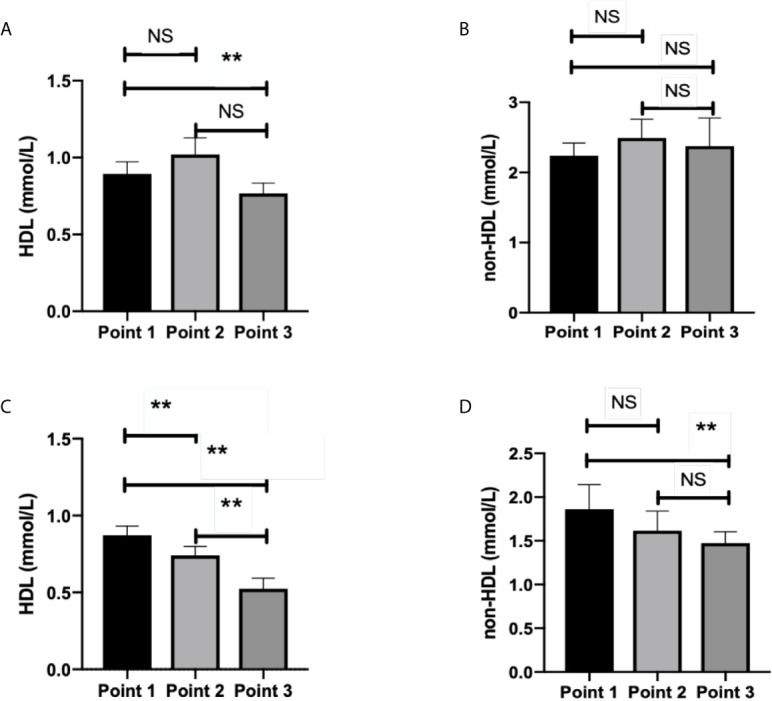
HDL level and non-HDL level in surviving and deceased patients. **(A)** shows that HDL level significantly decreased at time point 3 than that at time point 1 (*p*=0.007) in survived patients. No statistical differences existed between other time points (*p*>0.05) in terms of the HDL level of surviving patients. **(B)** shows no statistical differences among three time-points (*p*>0.05) in terms of non-HDL level of survived patients. **(C)** shows that statistical differences existed among three time-points (*p*<0.05) in terms of the HDL level of deceased patients. **(D)** showed that non-HDL level significantly decreased at time point 3 than at time point 1 (*p*=0.016) in deceased patients. No statistical differences existed between other time-points in terms of the non-HDL level of deceased patients (*p* > 0.05). Point 1: on admission day; Point 2: day 2-3 after the admission; Point 3: last test before achievement of clinical outcome. NS: *p* > 0.05; **: *p* < 0.01.

HDL level significantly decreased at TP-3 compared with TP-1 in group 2 (*p*=0.007, **Figure 2A**). There were no statistical differences among three time-points in terms of the non-HDL level of group 2 (*p*>0.05, **Figure 2B**). In group 1, significant differences in HDL level were observed among the three time-points (*p*<0.01, **Figure 2C**). Non-HDL level significantly decreased at TP-3 than that at TP-1 (*p*=0.016, **Figure 2D**).

### The Level of HDL-c and TC as the Predictors of In-Hospital Mortality in COVID-19 Critical Patients

The potential explanatory variables associated with in-hospital mortality included clinical demographic factors, laboratory profiles listed in **Tables 1**, **2**. Logistic regression analysis revealed that TC level at TP-2 (Exp[B]=0.311, 95%CI=0.104-0.930, *P*=0.037), HDL-c level both at TP-1 (Exp[B]=0.022, 95%CI=0.001-0.482, *P*=0.016) and TP-3 (Exp[B]=0.039, 95%CI=0.001-1.038, *P*=0.029) were the independent predictors of in-hospital mortality respectively (**Table 4**).

**Table 4:** Univariate and multivariate analysis for factors associated with in-hospital mortality.

	Univariate			Multivariate		
	*P* value	Exp (B)	95% CI	*P* value	Exp (B)	95% CI
In-hospital mortality						
HDL-c^a^*	0.021	0.033	0.002-0.601	0.016	0.022	0.001-0.482
LDL-c^a^	0.068	0.381	0.135-1.075			
D-dimer^a^	0.051	1.053	1.000-1.008			
CHOL^b^	0.034	0.301	0.099-0.911	0.037	0.311	0.104-0.930
LDL-c^b^	0.089	0.158	0.019-1.326			
Alb^b^	0.049	0.748	0.561-0.998			
HDL-c^c^*	0.025	0.032	0.001-1.022	0.029	0.039	0.001-1.038
LDL-c^c^	0.090	1.002	1.000-1.003			
Alb^c^	0.070	0.847	0.708-1.014			

HDL, high density lipoprotein; LDL, low density lipoprotein; CHOL, total cholesterol; Alb, Albumin.a, on admission; b, day 2-3 after admission; c, last test before achievement of clinical outcome.*Adjusted for age and gender.

## Discussion

Our observational study showed that abnormalities of lipid metabolism existed in the critical patients with COVID-19. LDL-c, HDL-c, ApoA, and Apo B levels decreased steadily from hospital admission to the end of clinical outcome in critically ill Covid-19 patients. We also found that HDL-c level both on admission day and the day just before achievement of clinical outcome, and TC level at 2-3days after admission were significantly associated with mortality. Nutrition is a key determinant of health and it is also part of the treatment strategies for acute and chronic diseases (12).

The role of cholesterol in immunity is increasingly recognized in multiple observational studies. The lower level of LDL was linked to the higher prevalence of mortality and poor prognosis in patients with severe infections (13, 14). It has been suggested that low cholesterol levels could be regarded as a marker for a worse prognosis in sepsis patients. Multiple studies have indicated that heterozygous hypolipidemia is associated with a decreased inflammatory response to infection and a higher risk for severe infections and sepsis (15). COVID-19 is closely related to negative outcomes in the elderly, with comorbidities and hypoalbuminemic patients (16). Meanwhile, a recent study also showed that a low prealbumin level could predict the progression of acute respiratory distress syndrome (ARDS) (16). Our results also showed that severe hypoalbuminemia existed in critical patients with COVID-19 at day 2-3 after admission and lower albumin level at last test before the achievement of clinical outcome in a non-survival patients group compared with that in survival cases. Therefore, the timing of nutritional intervention may be a major issue especially for those who had rapid progression and were immediately admitted to ICU (17).

Our study showed that TC level was significantly lower in the non-survival group compared to those in the survival group on admission, day 2-3 after admission as well as the last test before achievement of clinical outcome. HDL-c level and Apo A level were statistically lower in the non-survival group than those in the survival group on admission and the last test before achievement of clinical outcome. Similar events could also be found in terms of LDL-c level at the last test before the achievement of clinical outcome. Hypolipidemia may be a rare issue and is associated with a genetic disease or secondary elements, such as inflammation, malnutrition, drugs, and many others. Recently, one study showed that reduced lipid level in patients with COVID-19 was closely related to the severity of symptoms (18). Another observation study also indicated that HDL-c level remained relatively low during the treatment stage and after recovery in survival patients. While, the levels of LDL-c, HDL-c, and TC in non-survival patients decreased continuously (9). The role of HDL as an anti-inflammatory mediator in addition to its involvement in reverse cholesterol transfer is well established (19). Another study showed an inverse correlation between the levels of PCT and HDL, suggesting an inhibitory role of HDL on the inflammatory processes that stimulate PCT production.

Results also indicated that the degree of decreased LDL-c levels had high odds associated with the severity and mortality of COVID-19 (9). A lower level of HDL-c, TC, and LDL-c, together with other risk elements (16), such as higher d-dimer, older age, and poor glucose control (20), may provide great insight into the prognosis of COVID-19 at an early stage, especially in critical cases.

Lipid rafts containing numerous cholesterol are considered to play an important role in promoting viral infectivity (21). Lipid rafts are essential for the interaction between the S protein and ACE2 receptor as well as for facilitating the process of viral endocytosis (22). Cholesterol depletion impaired viral entry and virus-induced fusion, suggest that cholesterol is important during the post-binding stages (23). Although the above reported *in vitro* data suggest the essential role of lipid rafts and cholesterol in viral entry, specific confirmation *in vivo* is needed.

Total cholesterol levels in admitted COVID-19 patients can be extremely variable and time-dependent. A recent study revealed that COVID-19 patients had sharply decreased total cholesterol and low-density lipoprotein cholesterol (LDL-C) levels respectively (24). Although several mechanisms for the acute fall in cholesterol were suggested, it remains unclear whether these changes in serum cholesterol are related to viral–host cell fusion and entry (24). Therefore, the timing of cholesterol lowering may be fundamental in the management of critically unwell patients, and these therapies might be better suited earlier in the disease course prior to patients being admitted to critical care (25).

A number of retrospective studies have shown lower inflammatory parameters, decreased incidence of severe clinical manifestations, or reduced mortality rates in COVID-19 patients under statin treatment as compared to those not taking statins (6, 26). However, consistent evidence from prospective studies is not currently available. In addition, two recent meta-analyses of observational studies, exploring the impact of statin therapy on COVID-19 outcomes reported contrasting results (27, 28). Therefore, clinical trials investigating this issue are eagerly awaited.

The beneficial effects of statins as add-on therapy in COVID-19 may be raised when considering the possible detrimental impact of reduced low-density lipoprotein (LDL) cholesterol levels on COVID-19 prognosis, as suggested by some retrospective studies (9). However, reverse causality (i.e., viral infection as a cause of LDL cholesterol reduction) instead of causality (i.e., LDL cholesterol reduction as a factor promoting viral infection) might explain the association between LDL cholesterol and severe COVID-19 manifestations (29).

There are various possible interpretations for the aberrant lipid profiles in critical patients with COVID-19. First, patients may suffer from liver dysfunction and thereby the relative lipid biosynthesis was damaged. Although our data showed that serum levels of AST, ALT, or ALP statistically increased in the non-survival group compared with those in the survival group, it still needed to be determined whether liver dysfunction was closely associated with reduced lipid profiles such as LDL-c, HDL-c, and ApoA, Apo B. Second, the release of inflammatory cytokines induced by virus infection modulates lipid metabolism. Those cytokines, such as IL-6 and IL-1β, may alter liver function and diminish cholesterol efflux as well as transportation as so to manipulate lipid profiles (30). Moreover, increased free radicals in host cells infected by the virus may rapidly degrade lipids leading the decreased levels of LDL-c, HDL-c, and total cholesterol (31). Last but not the least, vascular permeability may be easily altered by virus infection in critical patients with COVID-19 so that exudates could be formed in tissues, such as alveolar spaces, accumulated by a series of leaked cholesterol particles. Exudative fluids could be explored in the early era of lung pathology with COVID-19 (32) and contain a high concentration of protein and cholesterol, caused by inflammation-associated vascular permeability (33). Therefore, it is noteworthy that dyslipidemia plays a crucial role in the pathological development of critical COVID-19 and urgent investigation of the underlying mechanisms should be launched.

The limitations of this study should be noted. First, this study is a pilot and retrospective analysis. Our preliminary results showed that deteriorative disorders in lipid metabolism may exist among the critical patients with COVID-19. Further prospective and large-scale studies with long-term follow-up are warranted. Second, before admission to ICU, patients were treated by various medical strategies which could interfere with the results. Meanwhile, given the urgent circumstances, the height and body weight of the patients were not accurately measured in all the critical patients with COVID-19. It is still unknown whether and how those elements might play a role in our data. Third, the number of critical cases was relatively small (N=48). Moreover, we only observed critical patients with COVID-19. A data set of patients with mild COVID-19 and severe cases in terms of lipid profiles should be conducted. Fourth, as this study indicates that dyslipidemia may be related to the progression of critical COVID-19, the relative mechanisms should be further explored in the future.

## Conclusions

The data indicated that dyslipidemia existed in the critical patients with COVID-19. HDL-c and TC levels may be served as important indicators of mortality. The findings shed light on the importance of lipid metabolism in the progression of critical patients with COVID-19. It may provide new insight into evaluating the prognosis of COVID-19.

## Data Availability Statement

The original contributions presented in the study are included in the article/supplementary material. Further inquiries can be directed to the corresponding authors.

## Ethics Statement

The studies involving human participants were reviewed and approved by Medical Ethics Committee of Renji Hospital, school of Medicine, Shanghai Jiaotong University (KY2020-105). The patients/participants provided their written informed consent to participate in this study.

## Author Contributions

JY, HX, YZ, L-ST, and WeiL drafted the article, and contributed to the conception and design of the work. HX, WenL, XH, QM, and SL contributed to data collection. JY, YZ, and L-ST contributed to the data analysis and interpretation. JY, JM, YZ, HX, WenL, XH, QM, SL, L-ST, and WeiL contributed to the revision of the article. All authors contributed to the article and approved the submitted version.

## Funding

This work is supported by the grant 2016YFC1305600, 2016YFC1305602 from the Major Chronic Non-communicable Disease Prevention and Control Research, National Key R&D Program of China.

## Conflict of Interest

The authors declare that the research was conducted in the absence of any commercial or financial relationships that could be construed as a potential conflict of interest.

## Abbreviations

T2DM, Type 2 diabetes mellitus; CVD, cardiovascular diseases; Cre: creatinine; UA, uric acid; Cys, Cystatin; PCT, Procalcitonin; ALT, Alanine aminotransferase; AST, Glutamic oxaloacetylase; TP, Total protein; ALB, Albumin; TBIL, total bilirubin; DBIL, Direct bilirubin; ALP, alkaline phosphatase; GGT, r-glutamyltranspeptidase; C-Ca2+ corrected calcium; LDH, lactate dehydrogenase; HBDH, α-Hydroxybutyrate dehydrogenase; TC, total cholesterol; TG, triglyceride; HDL, high density lipoprotein; LDL, low density lipoprotein; APOA, apolipoprotein A; APOB, apolipoprotein B.
